# Supplementary material for: Development and comparison of loop-mediated isothermal amplification with quantitative PCR for the specific detection of Saprolegnia spp
Source: PLoS One. 2021 Dec 13;16(12):e0250808. doi: 10.1371/journal.pone.0250808 (PMC8668100; doi:10.1371/journal.pone.0250808)
Supplement: Attachment — Submitted filename: Response to Reviewers.docx [file pone.0250808.s001.docx]

**PLOS ONE Response to Reviewers**

***Reviewer One***

Please use the space provided to explain your answers to the questions above. You may also include additional comments for the author, including concerns about dual publication, research ethics, or publication ethics. (Please upload your review as an attachment if it exceeds 20,000 characters)

Reviewer #1: The work is indeed important for all aquaculture pathogen surveillance projects.

Good work..Indeed a good manuscript to be published.

the real time data maybe should be made more clearer, such as no of technical and biological replicates..That is only one one reservation

***Additional Editor Comments (if provided):***

I got the recommendations and comments from an expert reviewer on the field. The both reviewers agree that the manuscript is technically sound and the data support the conclusions. However, lack of the real time data, such as no of technical and biological replicates. That is only one one reservation and I totally share their comments. Therefore, I can invite you to submit a revised version of the manuscript that addresses the major point raised by the reviewers.

**Response**

We thank the reviewers for their helpful comments and supportive message.

Regarding technical and biological replications in our qPCR experiment, we followed the guiding principles from Blainey, P., Krzywinski, M. & Altman, N. Replication. Nat Methods 11, 879–880 (2014). <https://doi.org/10.1038/nmeth.309>

We agree with the reviewers that having both technical and biological replication is of a great value. In our case, the qPCR data of *Saprolegnia salmonis* was relevant to assess the robustness and to quantify other Saprolegnia spp. isolates for future experiments. Additionally, *Saprolegnia salmonis* has been used as our major biological sample in optimization of the reactions. It was later used as a positive control in all our experiments. We hope you agree that data in Table 3 represents both technical and biological replicates for our experiments. We added this information in each of our experiments where appropriated. Thank you.

***Journal Requirements:***

When submitting your revision, we need you to address these additional requirements.

1. Please ensure that your manuscript meets PLOS ONE's style requirements, including those for file naming. The PLOS ONE style templates can be found at

<https://journals.plos.org/plosone/s/file?id=wjVg/PLOSOne_formatting_sample_main_body.pdf>

and <https://journals.plos.org/plosone/s/file?id=ba62/PLOSOne_formatting_sample_title_authors_affiliations.pdf>

Modified as suggested

2. Thank you for stating the following in the Acknowledgments Section of your manuscript:

“This research was funded by Ohio Sea Grant (10000960) and Bowling Green State University. We thank Gayathri Beligala for her contribution to the experiments. “

We note that you have provided additional information within the Acknowledgements Section that is not currently declared in your Funding Statement. Please note that funding information should not appear in the Acknowledgments section or other areas of your manuscript. We will only publish funding information present in the Funding Statement section of the online submission form.

Please remove any funding-related text from the manuscript and let us know how you would like to update your Funding Statement. Currently, your Funding Statement reads as follows:

“SG-Ohio Sea Grant (10000960), https://ohioseagrant.osu.edu/

VP-Ohio Sea Grant (10000960),

https://ohioseagrant.osu.edu/

The funders had no role in study design, data collection and analysis, decision to publish, or preparation of the manuscript.”

We removed the funding-related text from the manuscript as suggested

Please update the funding Statement as follows:

“SG-Ohio Sea Grant (60063782), https://ohioseagrant.osu.edu/

VP-Ohio Sea Grant (60063782),

https://ohioseagrant.osu.edu/

The funders had no role in study design, data collection and analysis, decision to publish, or preparation of the manuscript.
